# Supplementary material for: Energy landscape analysis and time-series clustering analysis of patient state multistability related to rheumatoid arthritis drug treatment: The KURAMA cohort study
Source: PLoS One. 2024 May 6;19(5):e0302308. doi: 10.1371/journal.pone.0302308 (PMC11073743; doi:10.1371/journal.pone.0302308)
Supplement: S1 Table — (DOCX) [file pone.0302308.s002.docx]

S1 Table. List of state transition probabilities of all 597 patients

| Patient | Frequency of “good stability” | Frequency of “poor stability” | Direct transition from “good stability” to “poor stability” | Direct transition from “poor stability” to “good stability” | Transition from “good stability” to “poor stability” | Transition from “poor stability” to “good stability” |
| --- | --- | --- | --- | --- | --- | --- |
| 1 | 0.833333 | 0.166667 | 0.083333 | 0.166667 | 0.083333 | 0.166667 |
| 2 | 1 | 0 | 0 | 0 | 0 | 0 |
| 3 | 0.416667 | 0.583333 | 0 | 0.083333 | 0 | 0.083333 |
| 4 | 1 | 0 | 0 | 0 | 0 | 0 |
| 5 | 0.583333 | 0.416667 | 0.166667 | 0.166667 | 0.166667 | 0.166667 |
| 6 | 1 | 0 | 0 | 0 | 0 | 0 |
| 7 | 0.25 | 0.75 | 0.083333 | 0.083333 | 0.083333 | 0.083333 |
| 8 | 1 | 0 | 0 | 0 | 0 | 0 |
| 9 | 0.916667 | 0.083333 | 0.083333 | 0.083333 | 0.083333 | 0.083333 |
| 10 | 0.666667 | 0.333333 | 0 | 0.083333 | 0 | 0.083333 |
| 11 | 1 | 0 | 0 | 0 | 0 | 0 |
| 12 | 0.916667 | 0.083333 | 0 | 0.083333 | 0 | 0.083333 |
| 13 | 0.75 | 0.25 | 0.166667 | 0.25 | 0.166667 | 0.25 |
| 14 | 1 | 0 | 0 | 0 | 0 | 0 |
| 15 | 0.916667 | 0.083333 | 0 | 0.083333 | 0 | 0.083333 |
| 16 | 0.833333 | 0.166667 | 0 | 0.083333 | 0 | 0.083333 |
| 17 | 0.75 | 0.25 | 0.083333 | 0.166667 | 0.083333 | 0.166667 |
| 18 | 0.25 | 0.75 | 0.166667 | 0.083333 | 0.166667 | 0.083333 |
| 19 | 0.583333 | 0.416667 | 0 | 0.083333 | 0 | 0.083333 |
| 20 | 0.5 | 0.5 | 0.166667 | 0.25 | 0.166667 | 0.25 |
| 21 | 0.916667 | 0.083333 | 0 | 0.083333 | 0 | 0.083333 |
| 22 | 0.833333 | 0.166667 | 0.083333 | 0.166667 | 0.083333 | 0.166667 |
| 23 | 0 | 1 | 0 | 0 | 0 | 0 |
| 24 | 0.583333 | 0.416667 | 0.083333 | 0.166667 | 0.083333 | 0.166667 |
| 25 | 0.916667 | 0.083333 | 0 | 0.083333 | 0 | 0.083333 |
| 26 | 0.583333 | 0.416667 | 0.25 | 0.166667 | 0.25 | 0.166667 |
| 27 | 0.416667 | 0.583333 | 0.166667 | 0.083333 | 0.166667 | 0.083333 |
| 28 | 0.75 | 0.25 | 0 | 0.083333 | 0 | 0.083333 |
| 29 | 0.583333 | 0.416667 | 0.166667 | 0.25 | 0.166667 | 0.25 |
| 30 | 1 | 0 | 0 | 0 | 0 | 0 |
| 31 | 0 | 1 | 0 | 0 | 0 | 0 |
| 32 | 0 | 1 | 0 | 0 | 0 | 0 |
| 33 | 0.833333 | 0.166667 | 0.083333 | 0.166667 | 0.083333 | 0.166667 |
| 34 | 0.666667 | 0.333333 | 0.083333 | 0.166667 | 0.083333 | 0.166667 |
| 35 | 0 | 1 | 0 | 0 | 0 | 0 |
| 36 | 0.666667 | 0.333333 | 0.083333 | 0.166667 | 0.083333 | 0.166667 |
| 37 | 0.75 | 0.25 | 0.083333 | 0.166667 | 0.083333 | 0.166667 |
| 38 | 1 | 0 | 0 | 0 | 0 | 0 |
| 39 | 0.916667 | 0.083333 | 0 | 0.083333 | 0 | 0.083333 |
| 40 | 0.833333 | 0.166667 | 0.166667 | 0.166667 | 0.166667 | 0.166667 |
| 41 | 0.083333 | 0.916667 | 0.083333 | 0 | 0.083333 | 0 |
| 42 | 0.5 | 0.5 | 0.083333 | 0.166667 | 0.083333 | 0.166667 |
| 43 | 1 | 0 | 0 | 0 | 0 | 0 |
| 44 | 0.916667 | 0.083333 | 0 | 0.083333 | 0 | 0.083333 |
| 45 | 1 | 0 | 0 | 0 | 0 | 0 |
| 46 | 0.666667 | 0.333333 | 0 | 0.083333 | 0 | 0.083333 |
| 47 | 1 | 0 | 0 | 0 | 0 | 0 |
| 48 | 0 | 1 | 0 | 0 | 0 | 0 |
| 49 | 0.916667 | 0.083333 | 0.083333 | 0.083333 | 0.083333 | 0.083333 |
| 50 | 0.75 | 0.25 | 0 | 0.083333 | 0 | 0.083333 |
| 51 | 0.083333 | 0.916667 | 0.083333 | 0.083333 | 0.083333 | 0.083333 |
| 52 | 1 | 0 | 0 | 0 | 0 | 0 |
| 53 | 0 | 1 | 0 | 0 | 0 | 0 |
| 54 | 0.916667 | 0.083333 | 0 | 0.083333 | 0 | 0.083333 |
| 55 | 0.416667 | 0.583333 | 0.083333 | 0.166667 | 0.083333 | 0.166667 |
| 56 | 1 | 0 | 0 | 0 | 0 | 0 |
| 57 | 0 | 1 | 0 | 0 | 0 | 0 |
| 58 | 0.916667 | 0.083333 | 0 | 0.083333 | 0 | 0.083333 |
| 59 | 0.75 | 0.25 | 0.083333 | 0.166667 | 0.083333 | 0.166667 |
| 60 | 0.833333 | 0.166667 | 0 | 0.083333 | 0 | 0.083333 |
| 61 | 0.333333 | 0.666667 | 0.083333 | 0.166667 | 0.083333 | 0.166667 |
| 62 | 0.75 | 0.25 | 0.166667 | 0.166667 | 0.166667 | 0.166667 |
| 63 | 0.25 | 0.75 | 0.166667 | 0.166667 | 0.166667 | 0.166667 |
| 64 | 0.083333 | 0.916667 | 0.083333 | 0.083333 | 0.083333 | 0.083333 |
| 65 | 0.75 | 0.25 | 0.083333 | 0.166667 | 0.083333 | 0.166667 |
| 66 | 0.416667 | 0.583333 | 0.166667 | 0.166667 | 0.166667 | 0.166667 |
| 67 | 0.833333 | 0.166667 | 0.083333 | 0.166667 | 0.083333 | 0.166667 |
| 68 | 0.166667 | 0.833333 | 0.166667 | 0.166667 | 0.166667 | 0.166667 |
| 69 | 0 | 1 | 0 | 0 | 0 | 0 |
| 70 | 0.916667 | 0.083333 | 0 | 0.083333 | 0 | 0.083333 |
| 71 | 0.5 | 0.5 | 0.25 | 0.166667 | 0.25 | 0.166667 |
| 72 | 0.833333 | 0.166667 | 0.166667 | 0.166667 | 0.166667 | 0.166667 |
| 73 | 0.166667 | 0.833333 | 0.083333 | 0.083333 | 0.083333 | 0.083333 |
| 74 | 0.166667 | 0.833333 | 0.083333 | 0.083333 | 0.083333 | 0.083333 |
| 75 | 0 | 1 | 0 | 0 | 0 | 0 |
| 76 | 0.083333 | 0.916667 | 0.083333 | 0.083333 | 0.083333 | 0.083333 |
| 77 | 0.916667 | 0.083333 | 0 | 0.083333 | 0 | 0.083333 |
| 78 | 0.166667 | 0.833333 | 0 | 0.083333 | 0 | 0.083333 |
| 79 | 0.75 | 0.25 | 0 | 0.083333 | 0 | 0.083333 |
| 80 | 0.083333 | 0.916667 | 0.083333 | 0.083333 | 0.083333 | 0.083333 |
| 81 | 0.25 | 0.75 | 0.166667 | 0.25 | 0.166667 | 0.25 |
| 82 | 0.833333 | 0.166667 | 0.083333 | 0.166667 | 0.083333 | 0.166667 |
| 83 | 0.333333 | 0.666667 | 0.166667 | 0.166667 | 0.166667 | 0.166667 |
| 84 | 1 | 0 | 0 | 0 | 0 | 0 |
| 85 | 0.75 | 0.25 | 0.166667 | 0.25 | 0.166667 | 0.25 |
| 86 | 0.916667 | 0.083333 | 0 | 0.083333 | 0 | 0.083333 |
| 87 | 0.916667 | 0.083333 | 0 | 0.083333 | 0 | 0.083333 |
| 88 | 0.333333 | 0.666667 | 0.166667 | 0.166667 | 0.166667 | 0.166667 |
| 89 | 0.5 | 0.5 | 0.083333 | 0.166667 | 0.083333 | 0.166667 |
| 90 | 0.583333 | 0.416667 | 0.333333 | 0.25 | 0.333333 | 0.25 |
| 91 | 0.75 | 0.25 | 0.166667 | 0.166667 | 0.166667 | 0.166667 |
| 92 | 0.416667 | 0.583333 | 0.083333 | 0.166667 | 0.083333 | 0.166667 |
| 93 | 0.916667 | 0.083333 | 0 | 0.083333 | 0 | 0.083333 |
| 94 | 1 | 0 | 0 | 0 | 0 | 0 |
| 95 | 0.333333 | 0.666667 | 0.25 | 0.166667 | 0.25 | 0.166667 |
| 96 | 0 | 1 | 0 | 0 | 0 | 0 |
| 97 | 0.833333 | 0.166667 | 0 | 0.083333 | 0 | 0.083333 |
| 98 | 0.166667 | 0.833333 | 0.083333 | 0 | 0.083333 | 0 |
| 99 | 0.75 | 0.25 | 0.166667 | 0.25 | 0.166667 | 0.25 |
| 100 | 0 | 1 | 0 | 0 | 0 | 0 |
| 101 | 0.916667 | 0.083333 | 0.083333 | 0.083333 | 0.083333 | 0.083333 |
| 102 | 1 | 0 | 0 | 0 | 0 | 0 |
| 103 | 0.833333 | 0.166667 | 0 | 0.083333 | 0 | 0.083333 |
| 104 | 0.083333 | 0.916667 | 0.083333 | 0.083333 | 0.083333 | 0.083333 |
| 105 | 0 | 1 | 0 | 0 | 0 | 0 |
| 106 | 0.166667 | 0.833333 | 0.083333 | 0 | 0.083333 | 0 |
| 107 | 0.666667 | 0.333333 | 0.25 | 0.333333 | 0.25 | 0.333333 |
| 108 | 0.5 | 0.5 | 0 | 0.083333 | 0 | 0.083333 |
| 109 | 0 | 1 | 0 | 0 | 0 | 0 |
| 110 | 0.75 | 0.25 | 0.083333 | 0.166667 | 0.083333 | 0.166667 |
| 111 | 0 | 1 | 0 | 0 | 0 | 0 |
| 112 | 1 | 0 | 0 | 0 | 0 | 0 |
| 113 | 0.833333 | 0.166667 | 0.083333 | 0.166667 | 0.083333 | 0.166667 |
| 114 | 0.916667 | 0.083333 | 0 | 0.083333 | 0 | 0.083333 |
| 115 | 0.916667 | 0.083333 | 0 | 0.083333 | 0 | 0.083333 |
| 116 | 1 | 0 | 0 | 0 | 0 | 0 |
| 117 | 0.75 | 0.25 | 0 | 0.083333 | 0 | 0.083333 |
| 118 | 0.583333 | 0.416667 | 0.083333 | 0.166667 | 0.083333 | 0.166667 |
| 119 | 1 | 0 | 0 | 0 | 0 | 0 |
| 120 | 0.416667 | 0.583333 | 0.083333 | 0.166667 | 0.083333 | 0.166667 |
| 121 | 0 | 1 | 0 | 0 | 0 | 0 |
| 122 | 0.166667 | 0.833333 | 0.083333 | 0.083333 | 0.083333 | 0.083333 |
| 123 | 0.75 | 0.25 | 0 | 0.083333 | 0 | 0.083333 |
| 124 | 0.333333 | 0.666667 | 0.083333 | 0.083333 | 0.083333 | 0.083333 |
| 125 | 0.333333 | 0.666667 | 0.25 | 0.25 | 0.25 | 0.25 |
| 126 | 0.083333 | 0.916667 | 0.083333 | 0 | 0.083333 | 0 |
| 127 | 0.083333 | 0.916667 | 0.083333 | 0.083333 | 0.083333 | 0.083333 |
| 128 | 0.166667 | 0.833333 | 0.166667 | 0.083333 | 0.166667 | 0.083333 |
| 129 | 0.166667 | 0.833333 | 0.083333 | 0.083333 | 0.083333 | 0.083333 |
| 130 | 0.916667 | 0.083333 | 0 | 0.083333 | 0 | 0.083333 |
| 131 | 0.916667 | 0.083333 | 0 | 0.083333 | 0 | 0.083333 |
| 132 | 1 | 0 | 0 | 0 | 0 | 0 |
| 133 | 0 | 1 | 0 | 0 | 0 | 0 |
| 134 | 0 | 1 | 0 | 0 | 0 | 0 |
| 135 | 0.916667 | 0.083333 | 0 | 0.083333 | 0 | 0.083333 |
| 136 | 0.833333 | 0.166667 | 0 | 0.083333 | 0 | 0.083333 |
| 137 | 0.833333 | 0.166667 | 0.083333 | 0.166667 | 0.083333 | 0.166667 |
| 138 | 0 | 1 | 0 | 0 | 0 | 0 |
| 139 | 0.833333 | 0.166667 | 0.166667 | 0.166667 | 0.166667 | 0.166667 |
| 140 | 0.75 | 0.25 | 0.083333 | 0.166667 | 0.083333 | 0.166667 |
| 141 | 0 | 1 | 0 | 0 | 0 | 0 |
| 142 | 0.916667 | 0.083333 | 0 | 0.083333 | 0 | 0.083333 |
| 143 | 0 | 1 | 0 | 0 | 0 | 0 |
| 144 | 0.166667 | 0.833333 | 0.166667 | 0.083333 | 0.166667 | 0.083333 |
| 145 | 1 | 0 | 0 | 0 | 0 | 0 |
| 146 | 0.166667 | 0.833333 | 0.166667 | 0.166667 | 0.166667 | 0.166667 |
| 147 | 0.583333 | 0.416667 | 0.083333 | 0.166667 | 0.083333 | 0.166667 |
| 148 | 0.916667 | 0.083333 | 0 | 0.083333 | 0 | 0.083333 |
| 149 | 0.75 | 0.25 | 0.083333 | 0.166667 | 0.083333 | 0.166667 |
| 150 | 0 | 1 | 0 | 0 | 0 | 0 |
| 151 | 0.25 | 0.75 | 0.166667 | 0.166667 | 0.166667 | 0.166667 |
| 152 | 1 | 0 | 0 | 0 | 0 | 0 |
| 153 | 1 | 0 | 0 | 0 | 0 | 0 |
| 154 | 0.916667 | 0.083333 | 0 | 0.083333 | 0 | 0.083333 |
| 155 | 0.916667 | 0.083333 | 0 | 0.083333 | 0 | 0.083333 |
| 156 | 0.583333 | 0.416667 | 0 | 0.083333 | 0 | 0.083333 |
| 157 | 0.916667 | 0.083333 | 0 | 0.083333 | 0 | 0.083333 |
| 158 | 0.666667 | 0.333333 | 0.166667 | 0.25 | 0.166667 | 0.25 |
| 159 | 0.083333 | 0.916667 | 0.083333 | 0.083333 | 0.083333 | 0.083333 |
| 160 | 0.916667 | 0.083333 | 0 | 0.083333 | 0 | 0.083333 |
| 161 | 0.083333 | 0.916667 | 0.083333 | 0.083333 | 0.083333 | 0.083333 |
| 162 | 0.833333 | 0.166667 | 0 | 0.083333 | 0 | 0.083333 |
| 163 | 1 | 0 | 0 | 0 | 0 | 0 |
| 164 | 0 | 1 | 0 | 0 | 0 | 0 |
| 165 | 0.75 | 0.25 | 0.166667 | 0.083333 | 0.166667 | 0.083333 |
| 166 | 0.833333 | 0.166667 | 0.083333 | 0.083333 | 0.083333 | 0.083333 |
| 167 | 0.916667 | 0.083333 | 0 | 0.083333 | 0 | 0.083333 |
| 168 | 1 | 0 | 0 | 0 | 0 | 0 |
| 169 | 0.5 | 0.5 | 0.25 | 0.25 | 0.25 | 0.25 |
| 170 | 1 | 0 | 0 | 0 | 0 | 0 |
| 171 | 0 | 1 | 0 | 0 | 0 | 0 |
| 172 | 0.916667 | 0.083333 | 0 | 0.083333 | 0 | 0.083333 |
| 173 | 0.5 | 0.5 | 0.083333 | 0.166667 | 0.083333 | 0.166667 |
| 174 | 0.083333 | 0.916667 | 0.083333 | 0 | 0.083333 | 0 |
| 175 | 0.75 | 0.25 | 0.083333 | 0.166667 | 0.083333 | 0.166667 |
| 176 | 0.333333 | 0.666667 | 0.083333 | 0.166667 | 0.083333 | 0.166667 |
| 177 | 0.916667 | 0.083333 | 0.083333 | 0.083333 | 0.083333 | 0.083333 |
| 178 | 0.25 | 0.75 | 0.083333 | 0.083333 | 0.083333 | 0.083333 |
| 179 | 1 | 0 | 0 | 0 | 0 | 0 |
| 180 | 0.083333 | 0.916667 | 0 | 0.083333 | 0 | 0 |
| 181 | 0.916667 | 0.083333 | 0.083333 | 0.083333 | 0.083333 | 0.083333 |
| 182 | 0.916667 | 0.083333 | 0 | 0.083333 | 0 | 0.083333 |
| 183 | 0 | 1 | 0 | 0 | 0 | 0 |
| 184 | 0 | 1 | 0 | 0 | 0 | 0 |
| 185 | 0.833333 | 0.166667 | 0 | 0.083333 | 0 | 0.083333 |
| 186 | 0.333333 | 0.666667 | 0 | 0.083333 | 0 | 0.083333 |
| 187 | 0.416667 | 0.583333 | 0.166667 | 0.25 | 0.166667 | 0.25 |
| 188 | 0.666667 | 0.333333 | 0.083333 | 0.083333 | 0.083333 | 0.083333 |
| 189 | 0.75 | 0.25 | 0.083333 | 0.083333 | 0.083333 | 0.083333 |
| 190 | 1 | 0 | 0 | 0 | 0 | 0 |
| 191 | 1 | 0 | 0 | 0 | 0 | 0 |
| 192 | 0.5 | 0.5 | 0.083333 | 0.166667 | 0.083333 | 0.166667 |
| 193 | 0.25 | 0.75 | 0.166667 | 0.166667 | 0.166667 | 0.166667 |
| 194 | 0.833333 | 0.166667 | 0.083333 | 0.166667 | 0.083333 | 0.166667 |
| 195 | 0.333333 | 0.666667 | 0.083333 | 0.083333 | 0.083333 | 0.083333 |
| 196 | 0.583333 | 0.416667 | 0.166667 | 0.25 | 0.166667 | 0.25 |
| 197 | 0.666667 | 0.333333 | 0.166667 | 0.166667 | 0.166667 | 0.166667 |
| 198 | 0.666667 | 0.333333 | 0.166667 | 0.166667 | 0.166667 | 0.166667 |
| 199 | 0.083333 | 0.916667 | 0.083333 | 0.083333 | 0.083333 | 0.083333 |
| 200 | 0 | 1 | 0 | 0 | 0 | 0 |
| 201 | 0.833333 | 0.166667 | 0.083333 | 0.166667 | 0.083333 | 0.166667 |
| 202 | 0.75 | 0.25 | 0.083333 | 0.083333 | 0.083333 | 0.083333 |
| 203 | 0.583333 | 0.416667 | 0 | 0.083333 | 0 | 0.083333 |
| 204 | 0.916667 | 0.083333 | 0 | 0.083333 | 0 | 0.083333 |
| 205 | 0 | 1 | 0 | 0 | 0 | 0 |
| 206 | 0 | 1 | 0 | 0 | 0 | 0 |
| 207 | 0 | 1 | 0 | 0 | 0 | 0 |
| 208 | 1 | 0 | 0 | 0 | 0 | 0 |
| 209 | 0.833333 | 0.166667 | 0.083333 | 0.166667 | 0.083333 | 0.166667 |
| 210 | 1 | 0 | 0 | 0 | 0 | 0 |
| 211 | 0.916667 | 0.083333 | 0.083333 | 0.083333 | 0.083333 | 0.083333 |
| 212 | 0.833333 | 0.166667 | 0.083333 | 0.166667 | 0.083333 | 0.166667 |
| 213 | 0.916667 | 0.083333 | 0 | 0.083333 | 0 | 0.083333 |
| 214 | 0.583333 | 0.416667 | 0 | 0.083333 | 0 | 0.083333 |
| 215 | 0.416667 | 0.583333 | 0.166667 | 0.166667 | 0.166667 | 0.166667 |
| 216 | 1 | 0 | 0 | 0 | 0 | 0 |
| 217 | 0.666667 | 0.333333 | 0.166667 | 0.166667 | 0.166667 | 0.166667 |
| 218 | 1 | 0 | 0 | 0 | 0 | 0 |
| 219 | 0.75 | 0.25 | 0.166667 | 0.25 | 0.166667 | 0.25 |
| 220 | 0.75 | 0.25 | 0.083333 | 0.166667 | 0.083333 | 0.166667 |
| 221 | 0.75 | 0.25 | 0 | 0.083333 | 0 | 0.083333 |
| 222 | 0.916667 | 0.083333 | 0 | 0.083333 | 0 | 0.083333 |
| 223 | 1 | 0 | 0 | 0 | 0 | 0 |
| 224 | 0.416667 | 0.583333 | 0.083333 | 0.083333 | 0.083333 | 0.083333 |
| 225 | 0.916667 | 0.083333 | 0 | 0.083333 | 0 | 0.083333 |
| 226 | 0.916667 | 0.083333 | 0 | 0.083333 | 0 | 0.083333 |
| 227 | 0.75 | 0.25 | 0.083333 | 0.083333 | 0.083333 | 0.083333 |
| 228 | 0.833333 | 0.166667 | 0.083333 | 0.083333 | 0.083333 | 0.083333 |
| 229 | 0.833333 | 0.166667 | 0.083333 | 0.166667 | 0.083333 | 0.166667 |
| 230 | 0.083333 | 0.916667 | 0 | 0.083333 | 0 | 0 |
| 231 | 0.75 | 0.25 | 0 | 0.083333 | 0 | 0.083333 |
| 232 | 0.583333 | 0.416667 | 0.25 | 0.333333 | 0.25 | 0.333333 |
| 233 | 0.75 | 0.25 | 0 | 0.083333 | 0 | 0.083333 |
| 234 | 0.083333 | 0.916667 | 0.083333 | 0.083333 | 0.083333 | 0.083333 |
| 235 | 0.166667 | 0.833333 | 0.083333 | 0.083333 | 0.083333 | 0.083333 |
| 236 | 1 | 0 | 0 | 0 | 0 | 0 |
| 237 | 0.75 | 0.25 | 0 | 0.083333 | 0 | 0.083333 |
| 238 | 1 | 0 | 0 | 0 | 0 | 0 |
| 239 | 0.833333 | 0.166667 | 0 | 0.083333 | 0 | 0.083333 |
| 240 | 1 | 0 | 0 | 0 | 0 | 0 |
| 241 | 1 | 0 | 0 | 0 | 0 | 0 |
| 242 | 1 | 0 | 0 | 0 | 0 | 0 |
| 243 | 0.75 | 0.25 | 0.166667 | 0.25 | 0.166667 | 0.25 |
| 244 | 0.666667 | 0.333333 | 0.083333 | 0.166667 | 0.083333 | 0.166667 |
| 245 | 0.166667 | 0.833333 | 0.166667 | 0.083333 | 0.166667 | 0.083333 |
| 246 | 0.416667 | 0.583333 | 0 | 0.083333 | 0 | 0.083333 |
| 247 | 0.166667 | 0.833333 | 0.083333 | 0.166667 | 0.083333 | 0.166667 |
| 248 | 0.916667 | 0.083333 | 0 | 0.083333 | 0 | 0.083333 |
| 249 | 0.166667 | 0.833333 | 0.083333 | 0.083333 | 0.083333 | 0.083333 |
| 250 | 0.416667 | 0.583333 | 0.166667 | 0.25 | 0.166667 | 0.25 |
| 251 | 0.833333 | 0.166667 | 0 | 0.083333 | 0 | 0.083333 |
| 252 | 0.916667 | 0.083333 | 0.083333 | 0.083333 | 0.083333 | 0.083333 |
| 253 | 0.083333 | 0.916667 | 0.083333 | 0.083333 | 0.083333 | 0.083333 |
| 254 | 1 | 0 | 0 | 0 | 0 | 0 |
| 255 | 0.75 | 0.25 | 0.166667 | 0.166667 | 0.166667 | 0.166667 |
| 256 | 0.666667 | 0.333333 | 0 | 0.083333 | 0 | 0.083333 |
| 257 | 1 | 0 | 0 | 0 | 0 | 0 |
| 258 | 0.083333 | 0.916667 | 0.083333 | 0.083333 | 0.083333 | 0.083333 |
| 259 | 0.833333 | 0.166667 | 0 | 0.083333 | 0 | 0.083333 |
| 260 | 1 | 0 | 0 | 0 | 0 | 0 |
| 261 | 0.833333 | 0.166667 | 0.083333 | 0.166667 | 0.083333 | 0.166667 |
| 262 | 0.75 | 0.25 | 0.083333 | 0.166667 | 0.083333 | 0.166667 |
| 263 | 0.833333 | 0.166667 | 0 | 0.083333 | 0 | 0.083333 |
| 264 | 0.75 | 0.25 | 0 | 0.083333 | 0 | 0.083333 |
| 265 | 0.083333 | 0.916667 | 0.083333 | 0.083333 | 0.083333 | 0.083333 |
| 266 | 0.916667 | 0.083333 | 0 | 0.083333 | 0 | 0.083333 |
| 267 | 0.833333 | 0.166667 | 0.083333 | 0.166667 | 0.083333 | 0.166667 |
| 268 | 1 | 0 | 0 | 0 | 0 | 0 |
| 269 | 0.916667 | 0.083333 | 0 | 0.083333 | 0 | 0.083333 |
| 270 | 0 | 1 | 0 | 0 | 0 | 0 |
| 271 | 1 | 0 | 0 | 0 | 0 | 0 |
| 272 | 0 | 1 | 0 | 0 | 0 | 0 |
| 273 | 0.25 | 0.75 | 0.083333 | 0.083333 | 0.083333 | 0.083333 |
| 274 | 0.166667 | 0.833333 | 0.083333 | 0.083333 | 0.083333 | 0.083333 |
| 275 | 0 | 1 | 0 | 0 | 0 | 0 |
| 276 | 1 | 0 | 0 | 0 | 0 | 0 |
| 277 | 0.333333 | 0.666667 | 0 | 0.083333 | 0 | 0.083333 |
| 278 | 0.083333 | 0.916667 | 0.083333 | 0.083333 | 0.083333 | 0.083333 |
| 279 | 0.166667 | 0.833333 | 0.083333 | 0.083333 | 0.083333 | 0.083333 |
| 280 | 0 | 1 | 0 | 0 | 0 | 0 |
| 281 | 0.916667 | 0.083333 | 0 | 0.083333 | 0 | 0.083333 |
| 282 | 0 | 1 | 0 | 0 | 0 | 0 |
| 283 | 0 | 1 | 0 | 0 | 0 | 0 |
| 284 | 0.5 | 0.5 | 0.166667 | 0.166667 | 0.166667 | 0.166667 |
| 285 | 0 | 1 | 0 | 0 | 0 | 0 |
| 286 | 0.916667 | 0.083333 | 0.083333 | 0.083333 | 0.083333 | 0.083333 |
| 287 | 0.916667 | 0.083333 | 0 | 0.083333 | 0 | 0.083333 |
| 288 | 0.916667 | 0.083333 | 0 | 0.083333 | 0 | 0.083333 |
| 289 | 0.333333 | 0.666667 | 0.083333 | 0.083333 | 0.083333 | 0.083333 |
| 290 | 0.166667 | 0.833333 | 0.083333 | 0.083333 | 0.083333 | 0.083333 |
| 291 | 0.916667 | 0.083333 | 0.083333 | 0 | 0 | 0 |
| 292 | 0 | 1 | 0 | 0 | 0 | 0 |
| 293 | 0.166667 | 0.833333 | 0.166667 | 0.166667 | 0.166667 | 0.166667 |
| 294 | 0.083333 | 0.916667 | 0.083333 | 0.083333 | 0.083333 | 0.083333 |
| 295 | 0.333333 | 0.666667 | 0.25 | 0.25 | 0.25 | 0.25 |
| 296 | 0.583333 | 0.416667 | 0.166667 | 0.166667 | 0.166667 | 0.166667 |
| 297 | 0.916667 | 0.083333 | 0 | 0.083333 | 0 | 0.083333 |
| 298 | 0.583333 | 0.416667 | 0.166667 | 0.166667 | 0.166667 | 0.166667 |
| 299 | 0.083333 | 0.916667 | 0.083333 | 0.083333 | 0.083333 | 0.083333 |
| 300 | 0.75 | 0.25 | 0.083333 | 0.166667 | 0.083333 | 0.166667 |
| 301 | 0.833333 | 0.166667 | 0.083333 | 0.083333 | 0.083333 | 0.083333 |
| 302 | 1 | 0 | 0 | 0 | 0 | 0 |
| 303 | 0.916667 | 0.083333 | 0.083333 | 0.083333 | 0.083333 | 0.083333 |
| 304 | 0 | 1 | 0 | 0 | 0 | 0 |
| 305 | 0.166667 | 0.833333 | 0.166667 | 0.166667 | 0.166667 | 0.166667 |
| 306 | 0.75 | 0.25 | 0.083333 | 0.166667 | 0.083333 | 0.166667 |
| 307 | 0.666667 | 0.333333 | 0.25 | 0.333333 | 0.25 | 0.333333 |
| 308 | 0.916667 | 0.083333 | 0 | 0.083333 | 0 | 0.083333 |
| 309 | 0 | 1 | 0 | 0 | 0 | 0 |
| 310 | 0.666667 | 0.333333 | 0.166667 | 0.166667 | 0.166667 | 0.166667 |
| 311 | 1 | 0 | 0 | 0 | 0 | 0 |
| 312 | 0 | 1 | 0 | 0 | 0 | 0 |
| 313 | 0.083333 | 0.916667 | 0.083333 | 0.083333 | 0.083333 | 0.083333 |
| 314 | 0.833333 | 0.166667 | 0.083333 | 0.166667 | 0.083333 | 0.166667 |
| 315 | 0 | 1 | 0 | 0 | 0 | 0 |
| 316 | 0.083333 | 0.916667 | 0.083333 | 0.083333 | 0.083333 | 0.083333 |
| 317 | 0.166667 | 0.833333 | 0.083333 | 0.083333 | 0.083333 | 0.083333 |
| 318 | 0.916667 | 0.083333 | 0 | 0.083333 | 0 | 0.083333 |
| 319 | 0 | 1 | 0 | 0 | 0 | 0 |
| 320 | 0.25 | 0.75 | 0.166667 | 0.25 | 0.166667 | 0.25 |
| 321 | 0.916667 | 0.083333 | 0 | 0.083333 | 0 | 0.083333 |
| 322 | 1 | 0 | 0 | 0 | 0 | 0 |
| 323 | 1 | 0 | 0 | 0 | 0 | 0 |
| 324 | 1 | 0 | 0 | 0 | 0 | 0 |
| 325 | 0.583333 | 0.416667 | 0.25 | 0.333333 | 0.25 | 0.333333 |
| 326 | 0.333333 | 0.666667 | 0.083333 | 0.166667 | 0.083333 | 0.166667 |
| 327 | 0.416667 | 0.583333 | 0.083333 | 0.083333 | 0.083333 | 0.083333 |
| 328 | 0.083333 | 0.916667 | 0.083333 | 0.083333 | 0.083333 | 0.083333 |
| 329 | 1 | 0 | 0 | 0 | 0 | 0 |
| 330 | 0.083333 | 0.916667 | 0.083333 | 0.083333 | 0.083333 | 0.083333 |
| 331 | 0 | 1 | 0 | 0 | 0 | 0 |
| 332 | 0.75 | 0.25 | 0.083333 | 0.166667 | 0.083333 | 0.166667 |
| 333 | 0.666667 | 0.333333 | 0.166667 | 0.25 | 0.166667 | 0.25 |
| 334 | 0 | 1 | 0 | 0 | 0 | 0 |
| 335 | 0.083333 | 0.916667 | 0 | 0.083333 | 0 | 0 |
| 336 | 0.083333 | 0.916667 | 0.083333 | 0.083333 | 0.083333 | 0.083333 |
| 337 | 1 | 0 | 0 | 0 | 0 | 0 |
| 338 | 0.25 | 0.75 | 0.25 | 0.25 | 0.25 | 0.25 |
| 339 | 0.083333 | 0.916667 | 0 | 0.083333 | 0 | 0 |
| 340 | 0.916667 | 0.083333 | 0 | 0.083333 | 0 | 0.083333 |
| 341 | 0 | 1 | 0 | 0 | 0 | 0 |
| 342 | 0.833333 | 0.166667 | 0 | 0.083333 | 0 | 0.083333 |
| 343 | 0.083333 | 0.916667 | 0.083333 | 0.083333 | 0.083333 | 0.083333 |
| 344 | 0.083333 | 0.916667 | 0.083333 | 0.083333 | 0.083333 | 0.083333 |
| 345 | 0.75 | 0.25 | 0.083333 | 0 | 0.083333 | 0 |
| 346 | 0.833333 | 0.166667 | 0 | 0.083333 | 0 | 0.083333 |
| 347 | 0 | 1 | 0 | 0 | 0 | 0 |
| 348 | 1 | 0 | 0 | 0 | 0 | 0 |
| 349 | 0.916667 | 0.083333 | 0.083333 | 0.083333 | 0.083333 | 0.083333 |
| 350 | 0.833333 | 0.166667 | 0.083333 | 0.166667 | 0.083333 | 0.166667 |
| 351 | 0.916667 | 0.083333 | 0 | 0.083333 | 0 | 0.083333 |
| 352 | 0.916667 | 0.083333 | 0 | 0.083333 | 0 | 0.083333 |
| 353 | 0.75 | 0.25 | 0.083333 | 0.166667 | 0.083333 | 0.166667 |
| 354 | 0.5 | 0.5 | 0.083333 | 0.083333 | 0.083333 | 0.083333 |
| 355 | 0.583333 | 0.416667 | 0.166667 | 0.25 | 0.166667 | 0.25 |
| 356 | 0.75 | 0.25 | 0 | 0.083333 | 0 | 0.083333 |
| 357 | 0.916667 | 0.083333 | 0 | 0.083333 | 0 | 0.083333 |
| 358 | 0.833333 | 0.166667 | 0.083333 | 0.166667 | 0.083333 | 0.166667 |
| 359 | 0.916667 | 0.083333 | 0 | 0.083333 | 0 | 0.083333 |
| 360 | 0.916667 | 0.083333 | 0.083333 | 0.083333 | 0.083333 | 0.083333 |
| 361 | 0.583333 | 0.416667 | 0.166667 | 0.25 | 0.166667 | 0.25 |
| 362 | 0.916667 | 0.083333 | 0 | 0.083333 | 0 | 0.083333 |
| 363 | 0.75 | 0.25 | 0 | 0.083333 | 0 | 0.083333 |
| 364 | 0.583333 | 0.416667 | 0 | 0.083333 | 0 | 0.083333 |
| 365 | 0.916667 | 0.083333 | 0 | 0.083333 | 0 | 0.083333 |
| 366 | 0 | 1 | 0 | 0 | 0 | 0 |
| 367 | 0.916667 | 0.083333 | 0 | 0.083333 | 0 | 0.083333 |
| 368 | 1 | 0 | 0 | 0 | 0 | 0 |
| 369 | 0 | 1 | 0 | 0 | 0 | 0 |
| 370 | 0.583333 | 0.416667 | 0 | 0.083333 | 0 | 0.083333 |
| 371 | 0.333333 | 0.666667 | 0.25 | 0.333333 | 0.25 | 0.333333 |
| 372 | 0.25 | 0.75 | 0.083333 | 0.083333 | 0.083333 | 0.083333 |
| 373 | 0.083333 | 0.916667 | 0.083333 | 0.083333 | 0.083333 | 0.083333 |
| 374 | 0.416667 | 0.583333 | 0.083333 | 0.166667 | 0.083333 | 0.166667 |
| 375 | 0.583333 | 0.416667 | 0.083333 | 0.166667 | 0.083333 | 0.166667 |
| 376 | 0 | 1 | 0 | 0 | 0 | 0 |
| 377 | 0.75 | 0.25 | 0.166667 | 0.25 | 0.166667 | 0.25 |
| 378 | 0 | 1 | 0 | 0 | 0 | 0 |
| 379 | 0.916667 | 0.083333 | 0 | 0.083333 | 0 | 0.083333 |
| 380 | 0.916667 | 0.083333 | 0.083333 | 0.083333 | 0.083333 | 0.083333 |
| 381 | 0.666667 | 0.333333 | 0.083333 | 0.166667 | 0.083333 | 0.166667 |
| 382 | 0.833333 | 0.166667 | 0.083333 | 0.166667 | 0.083333 | 0.166667 |
| 383 | 0.25 | 0.75 | 0.166667 | 0.083333 | 0.166667 | 0.083333 |
| 384 | 0.333333 | 0.666667 | 0 | 0.083333 | 0 | 0.083333 |
| 385 | 0.583333 | 0.416667 | 0.166667 | 0.166667 | 0.166667 | 0.166667 |
| 386 | 1 | 0 | 0 | 0 | 0 | 0 |
| 387 | 0.75 | 0.25 | 0.166667 | 0.25 | 0.166667 | 0.25 |
| 388 | 0.916667 | 0.083333 | 0 | 0.083333 | 0 | 0.083333 |
| 389 | 0.833333 | 0.166667 | 0 | 0.083333 | 0 | 0.083333 |
| 390 | 1 | 0 | 0 | 0 | 0 | 0 |
| 391 | 0.416667 | 0.583333 | 0.166667 | 0.25 | 0.166667 | 0.25 |
| 392 | 0.25 | 0.75 | 0.166667 | 0.166667 | 0.166667 | 0.166667 |
| 393 | 0.75 | 0.25 | 0.083333 | 0.166667 | 0.083333 | 0.166667 |
| 394 | 0.25 | 0.75 | 0.166667 | 0.166667 | 0.166667 | 0.166667 |
| 395 | 0.5 | 0.5 | 0.166667 | 0.25 | 0.166667 | 0.25 |
| 396 | 0.75 | 0.25 | 0.166667 | 0.166667 | 0.166667 | 0.166667 |
| 397 | 0.916667 | 0.083333 | 0 | 0.083333 | 0 | 0.083333 |
| 398 | 0.833333 | 0.166667 | 0.083333 | 0.083333 | 0.083333 | 0.083333 |
| 399 | 0.916667 | 0.083333 | 0 | 0.083333 | 0 | 0.083333 |
| 400 | 0.833333 | 0.166667 | 0 | 0.083333 | 0 | 0.083333 |
| 401 | 0.833333 | 0.166667 | 0.083333 | 0 | 0.083333 | 0 |
| 402 | 0.75 | 0.25 | 0.083333 | 0.166667 | 0.083333 | 0.166667 |
| 403 | 0.916667 | 0.083333 | 0.083333 | 0.083333 | 0.083333 | 0.083333 |
| 404 | 0.916667 | 0.083333 | 0 | 0.083333 | 0 | 0.083333 |
| 405 | 0.833333 | 0.166667 | 0.083333 | 0.166667 | 0.083333 | 0.166667 |
| 406 | 0.583333 | 0.416667 | 0.083333 | 0.083333 | 0.083333 | 0.083333 |
| 407 | 0.583333 | 0.416667 | 0.25 | 0.333333 | 0.25 | 0.333333 |
| 408 | 0 | 1 | 0 | 0 | 0 | 0 |
| 409 | 0.75 | 0.25 | 0.166667 | 0.25 | 0.166667 | 0.25 |
| 410 | 0 | 1 | 0 | 0 | 0 | 0 |
| 411 | 0.5 | 0.5 | 0.083333 | 0.166667 | 0.083333 | 0.166667 |
| 412 | 1 | 0 | 0 | 0 | 0 | 0 |
| 413 | 0 | 1 | 0 | 0 | 0 | 0 |
| 414 | 1 | 0 | 0 | 0 | 0 | 0 |
| 415 | 1 | 0 | 0 | 0 | 0 | 0 |
| 416 | 0.5 | 0.5 | 0.166667 | 0.25 | 0.166667 | 0.25 |
| 417 | 0.916667 | 0.083333 | 0 | 0.083333 | 0 | 0.083333 |
| 418 | 1 | 0 | 0 | 0 | 0 | 0 |
| 419 | 1 | 0 | 0 | 0 | 0 | 0 |
| 420 | 1 | 0 | 0 | 0 | 0 | 0 |
| 421 | 0.416667 | 0.583333 | 0.083333 | 0.166667 | 0.083333 | 0.166667 |
| 422 | 0.083333 | 0.916667 | 0.083333 | 0.083333 | 0.083333 | 0.083333 |
| 423 | 0.916667 | 0.083333 | 0 | 0.083333 | 0 | 0.083333 |
| 424 | 0.75 | 0.25 | 0 | 0.083333 | 0 | 0.083333 |
| 425 | 0.916667 | 0.083333 | 0 | 0.083333 | 0 | 0.083333 |
| 426 | 1 | 0 | 0 | 0 | 0 | 0 |
| 427 | 0.916667 | 0.083333 | 0 | 0.083333 | 0 | 0.083333 |
| 428 | 0.583333 | 0.416667 | 0 | 0.083333 | 0 | 0.083333 |
| 429 | 0.833333 | 0.166667 | 0.083333 | 0.166667 | 0.083333 | 0.166667 |
| 430 | 1 | 0 | 0 | 0 | 0 | 0 |
| 431 | 0.666667 | 0.333333 | 0 | 0.083333 | 0 | 0.083333 |
| 432 | 0.833333 | 0.166667 | 0.083333 | 0 | 0.083333 | 0 |
| 433 | 1 | 0 | 0 | 0 | 0 | 0 |
| 434 | 0.916667 | 0.083333 | 0.083333 | 0.083333 | 0.083333 | 0.083333 |
| 435 | 0.166667 | 0.833333 | 0 | 0.083333 | 0 | 0.083333 |
| 436 | 0.916667 | 0.083333 | 0 | 0.083333 | 0 | 0.083333 |
| 437 | 1 | 0 | 0 | 0 | 0 | 0 |
| 438 | 0.833333 | 0.166667 | 0.083333 | 0.083333 | 0.083333 | 0.083333 |
| 439 | 0.916667 | 0.083333 | 0.083333 | 0.083333 | 0.083333 | 0.083333 |
| 440 | 1 | 0 | 0 | 0 | 0 | 0 |
| 441 | 0 | 1 | 0 | 0 | 0 | 0 |
| 442 | 1 | 0 | 0 | 0 | 0 | 0 |
| 443 | 1 | 0 | 0 | 0 | 0 | 0 |
| 444 | 0.916667 | 0.083333 | 0.083333 | 0.083333 | 0.083333 | 0.083333 |
| 445 | 0.166667 | 0.833333 | 0.083333 | 0.166667 | 0.083333 | 0.166667 |
| 446 | 0 | 1 | 0 | 0 | 0 | 0 |
| 447 | 0 | 1 | 0 | 0 | 0 | 0 |
| 448 | 0.916667 | 0.083333 | 0 | 0.083333 | 0 | 0.083333 |
| 449 | 0.5 | 0.5 | 0.083333 | 0.166667 | 0.083333 | 0.166667 |
| 450 | 0.166667 | 0.833333 | 0.166667 | 0.166667 | 0.166667 | 0.166667 |
| 451 | 0.833333 | 0.166667 | 0 | 0.083333 | 0 | 0.083333 |
| 452 | 0.5 | 0.5 | 0.083333 | 0.166667 | 0.083333 | 0.166667 |
| 453 | 0.833333 | 0.166667 | 0.083333 | 0.166667 | 0.083333 | 0.166667 |
| 454 | 0.916667 | 0.083333 | 0 | 0.083333 | 0 | 0.083333 |
| 455 | 1 | 0 | 0 | 0 | 0 | 0 |
| 456 | 0.666667 | 0.333333 | 0.083333 | 0.166667 | 0.083333 | 0.166667 |
| 457 | 0.916667 | 0.083333 | 0.083333 | 0.083333 | 0.083333 | 0.083333 |
| 458 | 0.25 | 0.75 | 0.166667 | 0.166667 | 0.166667 | 0.166667 |
| 459 | 0.833333 | 0.166667 | 0.166667 | 0.166667 | 0.166667 | 0.166667 |
| 460 | 0.083333 | 0.916667 | 0 | 0.083333 | 0 | 0 |
| 461 | 0.916667 | 0.083333 | 0 | 0.083333 | 0 | 0.083333 |
| 462 | 0.833333 | 0.166667 | 0.083333 | 0.166667 | 0.083333 | 0.166667 |
| 463 | 1 | 0 | 0 | 0 | 0 | 0 |
| 464 | 0.166667 | 0.833333 | 0.083333 | 0.083333 | 0.083333 | 0.083333 |
| 465 | 0.916667 | 0.083333 | 0 | 0.083333 | 0 | 0.083333 |
| 466 | 0.5 | 0.5 | 0.083333 | 0.166667 | 0.083333 | 0.166667 |
| 467 | 0.333333 | 0.666667 | 0.083333 | 0.166667 | 0.083333 | 0.166667 |
| 468 | 0.75 | 0.25 | 0 | 0.083333 | 0 | 0.083333 |
| 469 | 0.416667 | 0.583333 | 0.166667 | 0.166667 | 0.166667 | 0.166667 |
| 470 | 0 | 1 | 0 | 0 | 0 | 0 |
| 471 | 1 | 0 | 0 | 0 | 0 | 0 |
| 472 | 0.083333 | 0.916667 | 0.083333 | 0 | 0.083333 | 0 |
| 473 | 0 | 1 | 0 | 0 | 0 | 0 |
| 474 | 0.416667 | 0.583333 | 0.166667 | 0.166667 | 0.166667 | 0.166667 |
| 475 | 0 | 1 | 0 | 0 | 0 | 0 |
| 476 | 0.916667 | 0.083333 | 0 | 0.083333 | 0 | 0.083333 |
| 477 | 0.75 | 0.25 | 0.083333 | 0.166667 | 0.083333 | 0.166667 |
| 478 | 0.833333 | 0.166667 | 0 | 0.083333 | 0 | 0.083333 |
| 479 | 0.333333 | 0.666667 | 0.083333 | 0.166667 | 0.083333 | 0.166667 |
| 480 | 0.25 | 0.75 | 0.083333 | 0.083333 | 0.083333 | 0.083333 |
| 481 | 0.333333 | 0.666667 | 0.083333 | 0.083333 | 0.083333 | 0.083333 |
| 482 | 0.666667 | 0.333333 | 0.166667 | 0.25 | 0.166667 | 0.25 |
| 483 | 0 | 1 | 0 | 0 | 0 | 0 |
| 484 | 0.25 | 0.75 | 0.166667 | 0.166667 | 0.166667 | 0.166667 |
| 485 | 1 | 0 | 0 | 0 | 0 | 0 |
| 486 | 1 | 0 | 0 | 0 | 0 | 0 |
| 487 | 1 | 0 | 0 | 0 | 0 | 0 |
| 488 | 0.916667 | 0.083333 | 0 | 0.083333 | 0 | 0.083333 |
| 489 | 1 | 0 | 0 | 0 | 0 | 0 |
| 490 | 0.666667 | 0.333333 | 0.083333 | 0.083333 | 0.083333 | 0.083333 |
| 491 | 0.333333 | 0.666667 | 0.083333 | 0.083333 | 0.083333 | 0.083333 |
| 492 | 0.083333 | 0.916667 | 0.083333 | 0.083333 | 0.083333 | 0.083333 |
| 493 | 0.083333 | 0.916667 | 0.083333 | 0.083333 | 0.083333 | 0.083333 |
| 494 | 0.75 | 0.25 | 0.166667 | 0.25 | 0.166667 | 0.25 |
| 495 | 0 | 1 | 0 | 0 | 0 | 0 |
| 496 | 0 | 1 | 0 | 0 | 0 | 0 |
| 497 | 0.916667 | 0.083333 | 0 | 0.083333 | 0 | 0.083333 |
| 498 | 0.833333 | 0.166667 | 0.166667 | 0.166667 | 0.166667 | 0.166667 |
| 499 | 0.166667 | 0.833333 | 0.083333 | 0.083333 | 0.083333 | 0.083333 |
| 500 | 0.666667 | 0.333333 | 0 | 0.083333 | 0 | 0.083333 |
| 501 | 0.5 | 0.5 | 0.25 | 0.333333 | 0.25 | 0.333333 |
| 502 | 1 | 0 | 0 | 0 | 0 | 0 |
| 503 | 0.833333 | 0.166667 | 0.166667 | 0.166667 | 0.166667 | 0.166667 |
| 504 | 0.083333 | 0.916667 | 0.083333 | 0.083333 | 0.083333 | 0.083333 |
| 505 | 0.833333 | 0.166667 | 0 | 0.083333 | 0 | 0.083333 |
| 506 | 0.916667 | 0.083333 | 0.083333 | 0.083333 | 0.083333 | 0.083333 |
| 507 | 0 | 1 | 0 | 0 | 0 | 0 |
| 508 | 0.333333 | 0.666667 | 0.25 | 0.166667 | 0.25 | 0.166667 |
| 509 | 0.333333 | 0.666667 | 0.25 | 0.25 | 0.25 | 0.25 |
| 510 | 0.916667 | 0.083333 | 0 | 0.083333 | 0 | 0.083333 |
| 511 | 0.666667 | 0.333333 | 0.083333 | 0.166667 | 0.083333 | 0.166667 |
| 512 | 0.666667 | 0.333333 | 0.166667 | 0.083333 | 0.166667 | 0.083333 |
| 513 | 0.583333 | 0.416667 | 0.166667 | 0.25 | 0.166667 | 0.25 |
| 514 | 1 | 0 | 0 | 0 | 0 | 0 |
| 515 | 1 | 0 | 0 | 0 | 0 | 0 |
| 516 | 0.833333 | 0.166667 | 0 | 0.083333 | 0 | 0.083333 |
| 517 | 0.916667 | 0.083333 | 0 | 0.083333 | 0 | 0.083333 |
| 518 | 1 | 0 | 0 | 0 | 0 | 0 |
| 519 | 0.916667 | 0.083333 | 0.083333 | 0.083333 | 0.083333 | 0.083333 |
| 520 | 1 | 0 | 0 | 0 | 0 | 0 |
| 521 | 0 | 1 | 0 | 0 | 0 | 0 |
| 522 | 0 | 1 | 0 | 0 | 0 | 0 |
| 523 | 0.833333 | 0.166667 | 0 | 0.083333 | 0 | 0.083333 |
| 524 | 0.833333 | 0.166667 | 0.083333 | 0.083333 | 0.083333 | 0.083333 |
| 525 | 0.666667 | 0.333333 | 0.083333 | 0.083333 | 0.083333 | 0.083333 |
| 526 | 0.083333 | 0.916667 | 0.083333 | 0.083333 | 0.083333 | 0.083333 |
| 527 | 0.666667 | 0.333333 | 0.166667 | 0.083333 | 0.166667 | 0.083333 |
| 528 | 0.5 | 0.5 | 0.083333 | 0 | 0.083333 | 0 |
| 529 | 0.916667 | 0.083333 | 0 | 0.083333 | 0 | 0.083333 |
| 530 | 0.916667 | 0.083333 | 0 | 0.083333 | 0 | 0.083333 |
| 531 | 0.916667 | 0.083333 | 0 | 0.083333 | 0 | 0.083333 |
| 532 | 0.583333 | 0.416667 | 0.083333 | 0 | 0.083333 | 0 |
| 533 | 0.25 | 0.75 | 0.083333 | 0 | 0.083333 | 0 |
| 534 | 0.666667 | 0.333333 | 0 | 0.083333 | 0 | 0.083333 |
| 535 | 0 | 1 | 0 | 0 | 0 | 0 |
| 536 | 0.833333 | 0.166667 | 0.083333 | 0.083333 | 0.083333 | 0.083333 |
| 537 | 0.416667 | 0.583333 | 0.25 | 0.25 | 0.25 | 0.25 |
| 538 | 0.833333 | 0.166667 | 0.083333 | 0.166667 | 0.083333 | 0.166667 |
| 539 | 0.583333 | 0.416667 | 0 | 0.083333 | 0 | 0.083333 |
| 540 | 0 | 1 | 0 | 0 | 0 | 0 |
| 541 | 0.916667 | 0.083333 | 0 | 0.083333 | 0 | 0.083333 |
| 542 | 0.25 | 0.75 | 0 | 0.083333 | 0 | 0.083333 |
| 543 | 0 | 1 | 0 | 0 | 0 | 0 |
| 544 | 0.916667 | 0.083333 | 0 | 0.083333 | 0 | 0.083333 |
| 545 | 0 | 1 | 0 | 0 | 0 | 0 |
| 546 | 0.5 | 0.5 | 0 | 0.083333 | 0 | 0.083333 |
| 547 | 0.333333 | 0.666667 | 0.166667 | 0.166667 | 0.166667 | 0.166667 |
| 548 | 0.916667 | 0.083333 | 0 | 0.083333 | 0 | 0.083333 |
| 549 | 0.083333 | 0.916667 | 0.083333 | 0.083333 | 0.083333 | 0.083333 |
| 550 | 0.833333 | 0.166667 | 0 | 0.083333 | 0 | 0.083333 |
| 551 | 0 | 1 | 0 | 0 | 0 | 0 |
| 552 | 0 | 1 | 0 | 0 | 0 | 0 |
| 553 | 0.583333 | 0.416667 | 0.083333 | 0.083333 | 0.083333 | 0.083333 |
| 554 | 0.833333 | 0.166667 | 0.083333 | 0.166667 | 0.083333 | 0.166667 |
| 555 | 0.75 | 0.25 | 0 | 0.083333 | 0 | 0.083333 |
| 556 | 0.916667 | 0.083333 | 0 | 0.083333 | 0 | 0.083333 |
| 557 | 0.666667 | 0.333333 | 0.083333 | 0 | 0.083333 | 0 |
| 558 | 1 | 0 | 0 | 0 | 0 | 0 |
| 559 | 0.416667 | 0.583333 | 0.166667 | 0.166667 | 0.166667 | 0.166667 |
| 560 | 0.083333 | 0.916667 | 0.083333 | 0.083333 | 0.083333 | 0.083333 |
| 561 | 0.75 | 0.25 | 0.083333 | 0.166667 | 0.083333 | 0.166667 |
| 562 | 0.833333 | 0.166667 | 0 | 0.083333 | 0 | 0.083333 |
| 563 | 0.916667 | 0.083333 | 0.083333 | 0.083333 | 0.083333 | 0.083333 |
| 564 | 0.416667 | 0.583333 | 0 | 0.083333 | 0 | 0.083333 |
| 565 | 0 | 1 | 0 | 0 | 0 | 0 |
| 566 | 0.333333 | 0.666667 | 0.166667 | 0.166667 | 0.166667 | 0.166667 |
| 567 | 1 | 0 | 0 | 0 | 0 | 0 |
| 568 | 0.583333 | 0.416667 | 0.333333 | 0.25 | 0.333333 | 0.25 |
| 569 | 0 | 1 | 0 | 0 | 0 | 0 |
| 570 | 0.833333 | 0.166667 | 0.083333 | 0.083333 | 0.083333 | 0.083333 |
| 571 | 0.916667 | 0.083333 | 0 | 0.083333 | 0 | 0.083333 |
| 572 | 0.416667 | 0.583333 | 0.25 | 0.333333 | 0.25 | 0.333333 |
| 573 | 0 | 1 | 0 | 0 | 0 | 0 |
| 574 | 0.666667 | 0.333333 | 0.083333 | 0.166667 | 0.083333 | 0.166667 |
| 575 | 0.333333 | 0.666667 | 0.166667 | 0.166667 | 0.166667 | 0.166667 |
| 576 | 0 | 1 | 0 | 0 | 0 | 0 |
| 577 | 0.916667 | 0.083333 | 0.083333 | 0.083333 | 0.083333 | 0.083333 |
| 578 | 0.916667 | 0.083333 | 0 | 0.083333 | 0 | 0.083333 |
| 579 | 1 | 0 | 0 | 0 | 0 | 0 |
| 580 | 0.916667 | 0.083333 | 0 | 0.083333 | 0 | 0.083333 |
| 581 | 0 | 1 | 0 | 0 | 0 | 0 |
| 582 | 0.083333 | 0.916667 | 0.083333 | 0.083333 | 0.083333 | 0.083333 |
| 583 | 0.916667 | 0.083333 | 0 | 0.083333 | 0 | 0.083333 |
| 584 | 0.583333 | 0.416667 | 0.083333 | 0.166667 | 0.083333 | 0.166667 |
| 585 | 0.166667 | 0.833333 | 0.083333 | 0.083333 | 0.083333 | 0.083333 |
| 586 | 0.666667 | 0.333333 | 0.25 | 0.166667 | 0.25 | 0.166667 |
| 587 | 0.75 | 0.25 | 0.166667 | 0.25 | 0.166667 | 0.25 |
| 588 | 0 | 1 | 0 | 0 | 0 | 0 |
| 589 | 0.5 | 0.5 | 0.083333 | 0 | 0.083333 | 0 |
| 590 | 0.333333 | 0.666667 | 0 | 0.083333 | 0 | 0.083333 |
| 591 | 0.916667 | 0.083333 | 0 | 0.083333 | 0 | 0.083333 |
| 592 | 0.916667 | 0.083333 | 0 | 0.083333 | 0 | 0.083333 |
| 593 | 0.916667 | 0.083333 | 0 | 0.083333 | 0 | 0.083333 |
| 594 | 0.916667 | 0.083333 | 0.083333 | 0.083333 | 0.083333 | 0.083333 |
| 595 | 0.666667 | 0.333333 | 0.083333 | 0.166667 | 0.083333 | 0.166667 |
| 596 | 0.833333 | 0.166667 | 0.083333 | 0.166667 | 0.083333 | 0.166667 |
| 597 | 0.416667 | 0.583333 | 0.083333 | 0.166667 | 0.083333 | 0.166667 |
