# Supplementary material for: Energy landscape analysis and time-series clustering analysis of patient state multistability related to rheumatoid arthritis drug treatment: The KURAMA cohort study
Source: PLoS One. 2024 May 6;19(5):e0302308. doi: 10.1371/journal.pone.0302308 (PMC11073743; doi:10.1371/journal.pone.0302308)
Supplement: S1 Appendix — The vertical axis indicates high and low energy. For the horizontal axis, the central vertical line divides the groups according to whether they are likely to transition to good stability or to persist in poor stability, according to the energy landscape analysis, and the solid red vertical line indicates the energy threshold (i.e., -1.48). (DOCX) [file pone.0302308.s003.docx]

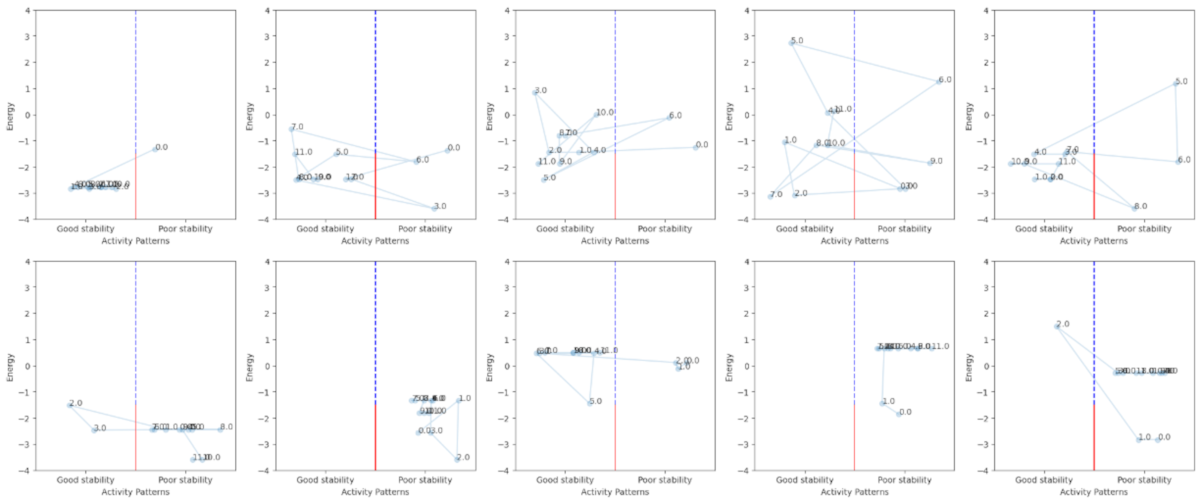


**S1 Appendix. The State Transitions of 10 Randomly Selected Individuals.** The vertical axis indicates high and low energy. For the horizontal axis, the central vertical line divides the groups according to whether they are likely to transition to good stability or to persist in poor stability, according to the energy landscape analysis, and the solid red vertical line indicates the energy threshold (i.e., -1.48).
