# Supplementary material for: Energy landscape analysis and time-series clustering analysis of patient state multistability related to rheumatoid arthritis drug treatment: The KURAMA cohort study
Source: PLoS One. 2024 May 6;19(5):e0302308. doi: 10.1371/journal.pone.0302308 (PMC11073743; doi:10.1371/journal.pone.0302308)
Supplement: S2 Appendix — Transitions in the good stability (A), poor stability (B), and unstable (C) clusters. Individuals in cluster 1 (poor stability) tend to have low energy and to remain in the poor stability quadrant. On the other hand, those in cluster 2 (unstable) generally have higher energy and tend to move between the good stability and poor stability quadrants. (DOCX) [file pone.0302308.s004.docx]

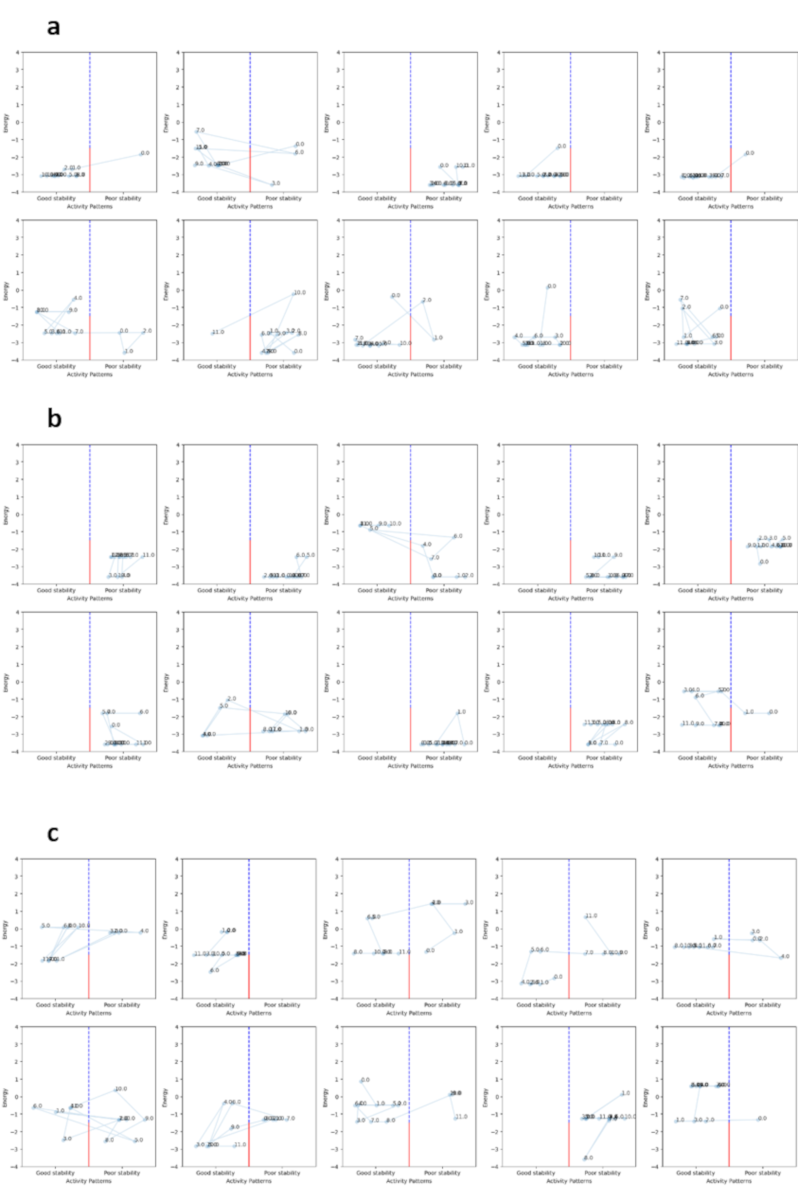
 **S2 Appendix. The State Transitions of 10 Randomly Selected Individuals from each Cluster.**

Transitions in the good stability (A), poor stability (B), and unstable (C) clusters. Individuals in cluster 1 (poor stability) tend to have low energy and to remain in the poor stability quadrant. On the other hand, those in cluster 2 (unstable) generally have higher energy and tend to move between the good stability and poor stability quadrants.
